# Overexpression of NIMA-related kinase 2 is associated with progression and poor prognosis of prostate cancer

**DOI:** 10.1186/s12894-015-0085-7

**Published:** 2015-08-29

**Authors:** Yan-Ru Zeng, Zhao-Dong Han, Cong Wang, Chao Cai, Ya-Qiang Huang, Hong-Wei Luo, Ze-Zhen Liu, Yang-Jia Zhuo, Qi-Shan Dai, Hai-Bo Zhao, Yu-Xiang Liang, Wei-De Zhong

**Affiliations:** Guangdong Provincial Institute of Nephrology, Southern Medical University, Guangzhou, 510515 China; Department of Urology, Guangdong Key Laboratory of Clinical Molecular Medicine and Diagnostics, Guangzhou First People’s Hospital, Guangzhou Medical University, Guangzhou, 510180 China; School of Pharmacy, Wenzhou Medical University, Wenzhou, 325035 China; Department of Urology, The Fifth Affiliated Hospital of Guangzhou Medical University, Guangzhou, 510799 China; Department of Urology, Huadu District People’s Hospital, Southern Medical University, Guangzhou, 510800 China; Urology Key Laboratory of Guangdong Province, The First Affiliated Hospital of Guangzhou Medical University, Guangzhou Medical University, Guangzhou, 510230 China; Department of Urology, Guangzhou First People’s Hospital, Guangzhou Medical University, Guangzhou, 510180 China

## Abstract

**Background:**

The NIMA-related kinase 2 (NEK2) is a serine/threonine kinase that is involved in regulation of centrosome duplication and spindle assembly during mitosis. Dysregulation of these processes causes chromosome instability and aneuploidy, which are hallmark changes of many solid tumors. However, whether aberrant expression of NEK2 is associated with outcome of prostate cancer (PCa) patients remains to be determined.

**Methods:**

Expression of NEK2 in human PCa cells and primary PCa tissues was assessed by quantitative RT-PCR. Expression of NEK2 in human PCa cells was depleted with siRNA. Effects of the depletion on cell proliferation, survival, and tumorigenicity were assessed both *in vitro* with cell cultures and *in vivo* with subcutaneous implantation of xenografts. *In silico* analyses of the online Taylor dataset were carried out to determine whether the expression level of NEK2 correlated with the clinicopathological characteristics of prostate cancer.

**Results:**

Compared with benign human prostatic epithelial cells and tissues, the expression of NEK2 was elevated in human PCa cells and primary PCa tissues. Depleting NEK2 expression inhibited human PCa cell proliferation *in vitro* and xenograft growth *in vivo*. Expression level of NEK2 in PCa positively correlated with the Gleason score and pathologic stage of the patient.

**Conclusion:**

The results suggest that overexpression of NEK2 has the potential to serve as a biomarker for PCa prognosis. Further validation with large sample pool is warrant.

## Background

The centrosome is the primary site of microtubule nucleation in cells, which plays a critical role in mitotic spindle formation and chromosome segregation [1, 2]. Normally, cells enter mitosis with two properly duplicated centrosomes. The process ensures bipolarity and correct axial positioning of the spindle. However, cancer cells often exhibit multipolar spindles associating with abnormal centrosome numbers or architectures [3–5]. A number of cell cycle-regulated protein kinases located at the centrosomes have been identified, which are required for mitotic progression and correct bipolar spindle formation [6]. Among them is NIMA-related kinase 2 (NEK2) that has three alternative splice isoforms (NEK2A, NEK2B and NEK2C). The expression of these three isoforms is tightly regulated in a cell cycle-dependent manner, suggesting that they may have isoform-specific roles in mitosis [7, 8].

NEK2 is a constitutive active serine and threonine kinase that phosphorylates multiple proteins involved in centrosome duplication and cell cycle regulation. It binds to microtubules and is enriched in the centrosome, where it contributes to centrosome splitting during the G2/M phase of the cell cycle [9]. Aberrant NEK2 activities cause failure in regulating centrosome duplication, resulting aneuploidy, and, therefore, are oncogenic [10]. Increased NEK2 expression causes premature splitting of centrosomes in human cells. Expression of a kinase-dead mutant of NEK2 induces centrosome abnormalities and aneuploidy [11, 12]. Similar to other kinases that are involved in spindle assembly or duplication, overexpression of NEK2 has been reported in several neoplastic diseases, such as preinvasive and invasive breast carcinomas [10], lung adenocarcinomas [13], testicular seminomas [14], and diffuse large B cell lymphomas [15]. Overexpression of NEK2 in non-transformed breast epithelial cells induces over duplication of the centrosome [10]. Increased expression of NEK2 causes centrosome amplification in breast cancer cells that express oncogenic K-RAS [16]. Furthermore, NEK2-dependent phosphorylation is required for kinetochore localizations of HEC1, a protein essential for faithful chromosome segregation [17]. In addition, NEK2 is also involved in regulation of viability and apoptosis. Depletion of NEK2 sensitizes HeLa cells to apoptosis and induces alternative splice of SRSF1 target genes [18].

Although a large number of data demonstrate elevated expression as well as reduced stability of NEK2 in cancer cells, whether NEK2 upregulation plays a role in PCa is not clearly. In this study, we investigated expression patterns of NEK2 in tissue microarrays (TMAs) of human PCa and the effect of NEK2 depletion on cell proliferation in PCa cells. The data showed strong correlation of NEK2 expression with the prognostic outcome of PCa patients. Together, our results indicate that NEK2 has a promising value for predicting outcome of PCa recurrence. Future studies with large sample pools are warranted to determine whether the expression level of NEK2 can serve as a biomarkers for PCa prognosis, which still needs prospective validation.

## Methods

### Patients and tissue samples

The study was approved by the Research Ethics Committee of Guangzhou First People’s Hospital, Guangzhou Medical University, China. An informed consent was obtained from all of the patients. All specimens were handled and remained anonymous according to the ethical and legal standards.

For immunohistochemistry analyses, TMAs with detail clinical information were purchased from Shanghai Outdo Biotech Co, LTD (Cat#: HPro-Ade180PG-01), which includes 99 primary PCa tissues and 81 adjacent non-cancerous prostate tissues from patients without chemotherapy or radiotherapy before surgery. The Taylor dataset (http://www.ncbi.nlm.nih.gov/geo/query/acc.cgi?acc=GSE21032) contains microarray data for mRNAs from 149 primary PCa tissues and 29 adjacent noncancerous prostate tissues [19]. The dataset contains patient survival time and follow-up examination between from 1 to 175 months with a median of 51 months post-surgery. The date of prostatectomy was considered as day 1 for the survival analyses. The PSA recurrence was defined as three successive PSA rises (final value >0.2 ng/ml), single PSA >0.4 ng/ml, or use of secondary therapy administered for detectable PSA >0.1 ng/ml, which was considered as the biochemical recurrence-free endpoint. The overall survival that was determined from the date of surgery to the time of the last follow-up or death.

### Cell culture, transfection, and treatment

For cell viability assays, human PCa LNCaP cells were seeded in 96-well plates (2 × 10^3^/well) and cultured for 24, 48, and 72 h. Cells were then incubated with 20 μl CCK-8 solution (Cat No: C0038, Beyotime, China) for 4 h at 37 °C. The absorbance was measured at the wavelength of 495 nm with a spectrophotometer. Data were expressed as mean ± SD of three independent samples. For RNA interference experiments, cells were transfected with siRNAs using Lipofectamine RNAiMAX (Invitrogen) according to manufacturer’s instructions and harvested at 24, 48, and 72 h after the transfection for protein and RNA analyses. Sequences of NEK2 siRNAs and scrambled siRNA are listed in Table 1.

**Table 1:** Sequences of the tested NEK2 siRNA targets

siRNA	mRNA target
Scr	ATAGTTCGTCCACTTCAGC
NEK2 .1	CGGAGGAAGAGTGATGGCAAGATAT
NEK2 .2	GAAGTGATGGTGGTCATACCGTATT
NEK2 .3	GCTGTATGAGTTATGTGCATTAATG

### Quantitative real-time RT-PCR

Total RNAs were isolated from cells and prostate tissues using the TRIzol reagent (Invitrogen, Carlsbad, CA, USA) according to the manufacturer’s instructions. Reverse transcription was carried out using the ReverTra Ace reagents kit (TOYOBO, Osaka, Japan). Real-time RT-PCR was carried out with the MiniOpticon real-time PCR detection system (Bio-Rad, Hercules, CA, USA) using the SYBR Green master mix (TaKaRa, Otsu, Japan). The thermal cycling conditions comprised of one cycle at 94 °C for 4 min, 40 cycles at 94 °C for 15 s, 60 °C for 15 s, and 72 °C for 15 s. All data were analyzed using the Opticon Monitor software (ver3.1, Bio-Rad). The expression of NEK2 was calculated as relative expression level to the GAPDH internal control using the comparative cycle threshold (CT) method. The primer sequences for NEK2 are GCTTAGGTAGCCCTTTTCATTTACA and GCCTCAGGTCTATGAACCCAG. The primer sequences for GAPDH are CATGGGTGTGAACCATGAGAAGT and ACAGTAGAGGCAGGGATGATGTTCT.

### Western blotting analysis

Proteins were extracted 48-hours post-transfection for Western blot analyses. Proteins (40 μg) were fractioned on SDS-PAGE and transferred onto Hybond nitrocellulose membranes (GE Healthcare). The membranes were blocked with 5 % skim milk in PBS-Tween 20 and probed with anti-NEK2 (bs-5732R, Bioss Co Ltd., China) or anti-β-actin antibody (sc-47778, Santa Cruz, USA). The specifically bound antibodies were detected with horse radish peroxidase-conjugated secondary antibody and visualized with the SuperSignal West PICO Chemiluminescent Detection Kit (Pierce Biotechnology). β-actin was used as an internal loading control.

### Tumor growth

All nude mice with an average age of 6–8 weeks were purchased from the Guangdong Medical Laboratory Animal Center. The NEK2 silenced cells were injected on the left side flank and the control cells on the right side flank. Briefly, 2×10^6^ cells were suspended in 0.1 ml culture medium, and then mixed with Matrigel (BD Biosciences,NO.356234) at the ratio of 1:1. The injections were carried out in a sterilized hood. Five mice per group were used and the experiments were repeated three times. The tumor size was measured with caliper at three time points and the means were calculated. The tumor tissues were harvested at day 43 after the implantation for histology and biochemistry analyses.

### Immunohistochemistry analysis

The specimens were fixed in 10 % neutral buffered formalin and subsequently embedded in paraffin. The paraffin-embedded tissues were sectioned at 4 μm thickness and then deparaffinized with xylene and rehydrated for subsequence analyses. For immunohistochemistry staining, the sections were subjected to a brief proteolytic digestion and a peroxidase blocking, the sections were incubated overnight with the primary antibody against NEK2 at a dilution of 1:400, at 4 °C. After washing, the sections were then incubated with peroxidase labeled polymer and substrate–chromogen from DAKO EnVision System (Dako Diagnostics, Switzerland) to detect the specifically bound antibodies as suggested by the manufacturer. No primary antibodies were used as the negative controls. The sections were then lightly counterstained with hematoxylin. The staining was scored by two independent experienced pathologists, who were blinded to the clinicopathological data and clinical outcomes of the patients. The scores of the two pathologists were compared. Any discrepant scores were trained through re-examining the staining by both pathologists to achieve a consensus score. The number of positive-staining cells in ten representative microscopic fields was counted, and the percentage of positive cells was calculated. The staining was subjected to arbitrative categorized to 5 groups based on the percentage of positive cells for semi-quantitative analyses as following: 0 (0 %), 1 (1–10 %), 2 (11–50 %), 3 (51–80 %) and 4 (>80 %). The staining intensity was visually scored and stratified as follows: 0 (negative), 1 (weak), 2 (moderate) and 3 (strong). A final immunoreactivity scores (IRS) were obtained for each case by adding the percentage and the intensity score [20, 21].

### Statistical analysis

The statistical analyses was carried out with the SPSS software (Version 13.0 for Windows, SPSS Inc., IL, USA) and SAS 9.1 (SAS Institute, Cary, NC, USA). Continuous variables were expressed as mean ± SD. The Kaplan–Meier method was used for the survival analyses and a log-rank test was used to analyze the difference of survival. The chiquest trend test was used for ordinal data analysis. Differences were considered statistically significant when *P* was smaller than 0.05.

## Results

### Depleting NEK2 expression reduces cell proliferation in PCa cells

**Fig. 1:**
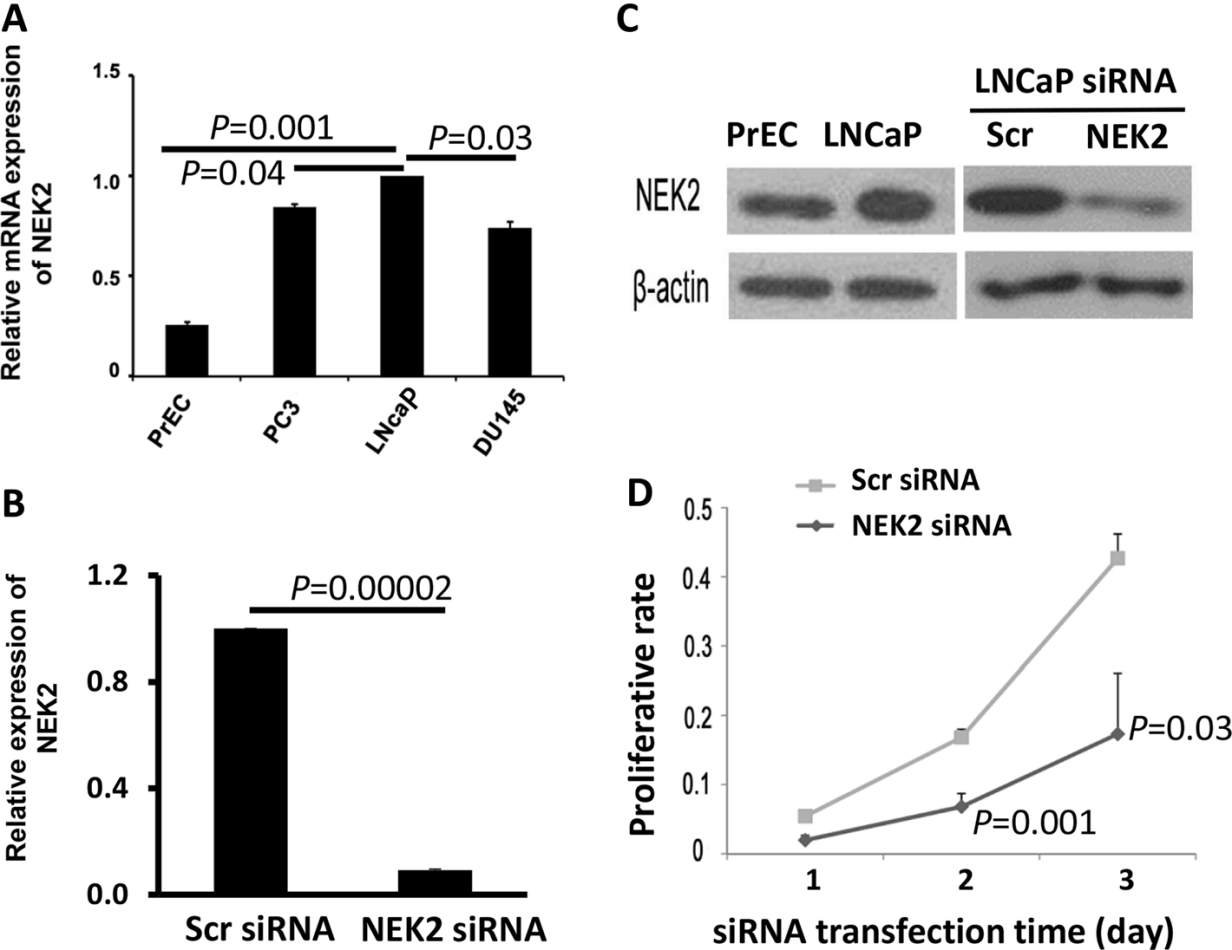
NEK2 expression and function in prostate epithelial cell lines. **a** & **b** Real-time RT-PCR analyses of NEK2 expression in benign human prostatic epithelial cells and PCa cells (**a**) or NEK2-depleted LNCaP cells. **c** Western blot analysis for NEK2 protein expression in PrEC and siRNA treated LNCaP cells. **d** Proliferation analyses of LNCaP cells with or without depletion of NEK2

To determine whether human PCa cells had aberrant NEK2 expression at the mRNA level, quantitative real-time RT-PCR was performed to assess the expression of NEK2 in three human PCa and one benign human prostatic epithelial cell lines. As shown in Fig. 1a, expression of NEK2 was higher in all three PCa cells than in benign human prostatic epithelial cells at the mRNA level, especially in LNCaP cell line. Then we chose LNCaP cells for the further study to determine whether high expression of NEK2 contributed to cell proliferation, siRNA was used to deplete the expression of NEK2 in LNCaP cells. Both real time RT-PCR and Western blot analyses showed that the expression of NEK2 was reduced at both mRNA and protein levels (Fig. 1b&c). Cell proliferation assays demonstrated that LNCaP cells with NEK2 depletion had a lower proliferation rate than the cells transfected with scramble siRNAs (Fig. 1d). The results suggest that high expression of NEK2 promotes cell proliferation.

### Depletion of NEK2 expression suppresses tumorigenicity of LNCaP cells

**Fig. 2:**
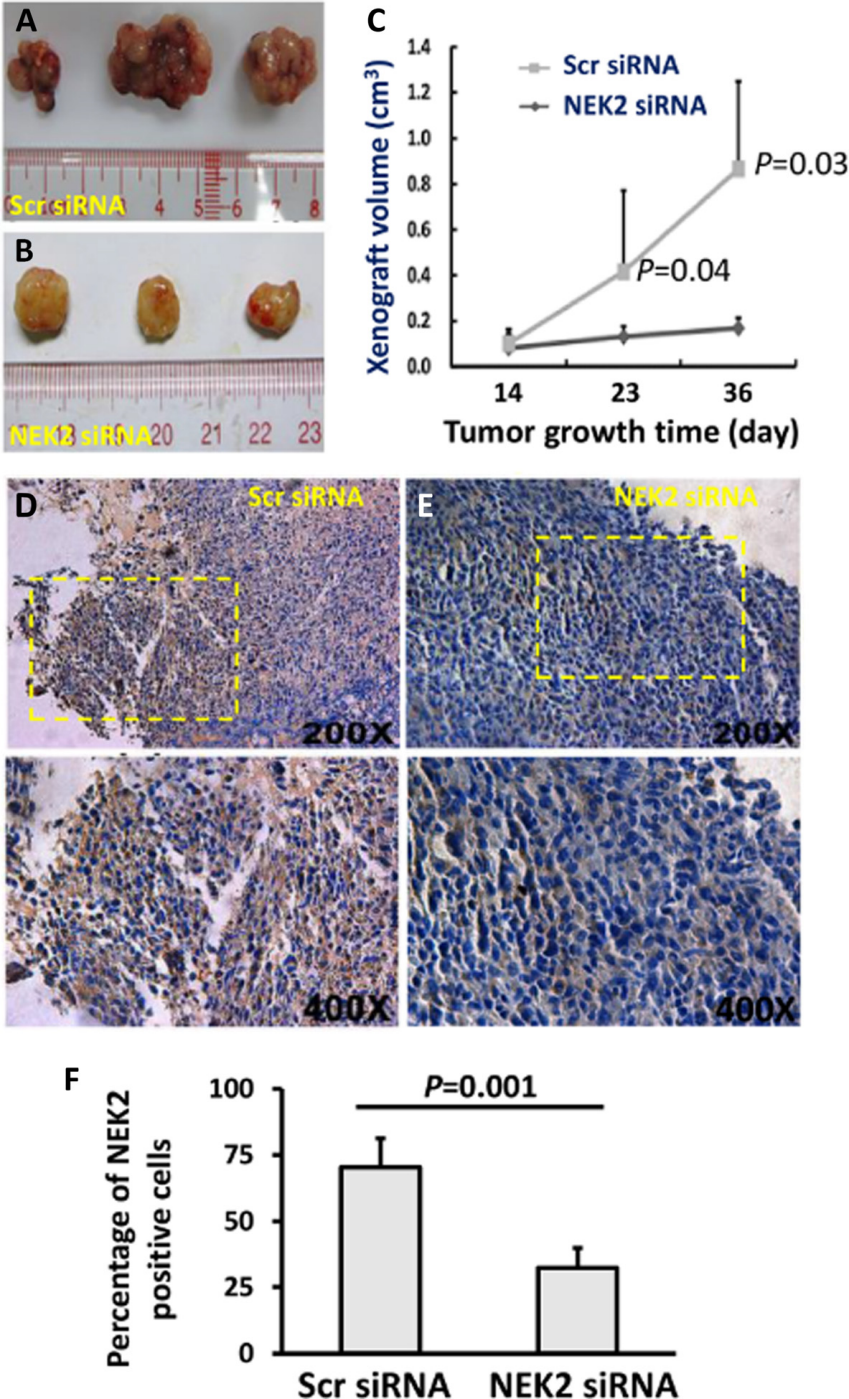
Depletion of NEK2 by siRNA inhibits the growth of LNCaP subcutaneous xenograft growth in nude mice. **a** & **b** Representative images of LNCaP xenografts. **c** Tumor growth curves of LNCaP xenografts. **d** & **e** Immunostaining of NEK2 in LNCaP xenografts. The figure magnification is × 200 and × 400, respectively. **f** Statistical analysis of the NEK2 positive cells in the immunostaining

To determine whether depletion of NEK2 affected the tumorigenicity of LNCaP cells, the silenced and control cells were implanted to the flanks of the same mice. The tumor sizes were measured at day 14, 23, and 36 post the implantation. It was apparent that tumors derived from NEK2 silenced cells were smaller than those from control cells (Fig. 2a, b&c). The tumors were harvested at day 43 after the implantation for further analyses. In addition to smaller size, the tumors with NEK2 silenced cells were relatively pale than the tumors from control cells (Fig. 2b). Immunostaining revealed that expression of NEK2 protein was reduced compared with the tumors derived from scramble siRNA transfected control cells. High magnification images showed that nuclear and cytosol staining of NEK2 staining was visible in control cells, but was absent in NEK2 depleted cells (Fig. 2d&e). The percentage of NEK2 positive cells was dramatically decreased in NEK2 siRNA group (Fig. 2f).

### The expression level of NEK2 is associated with the clinicopathological characteristics of PCa and tumor recurrence time of PCa patients

**Fig. 3:**
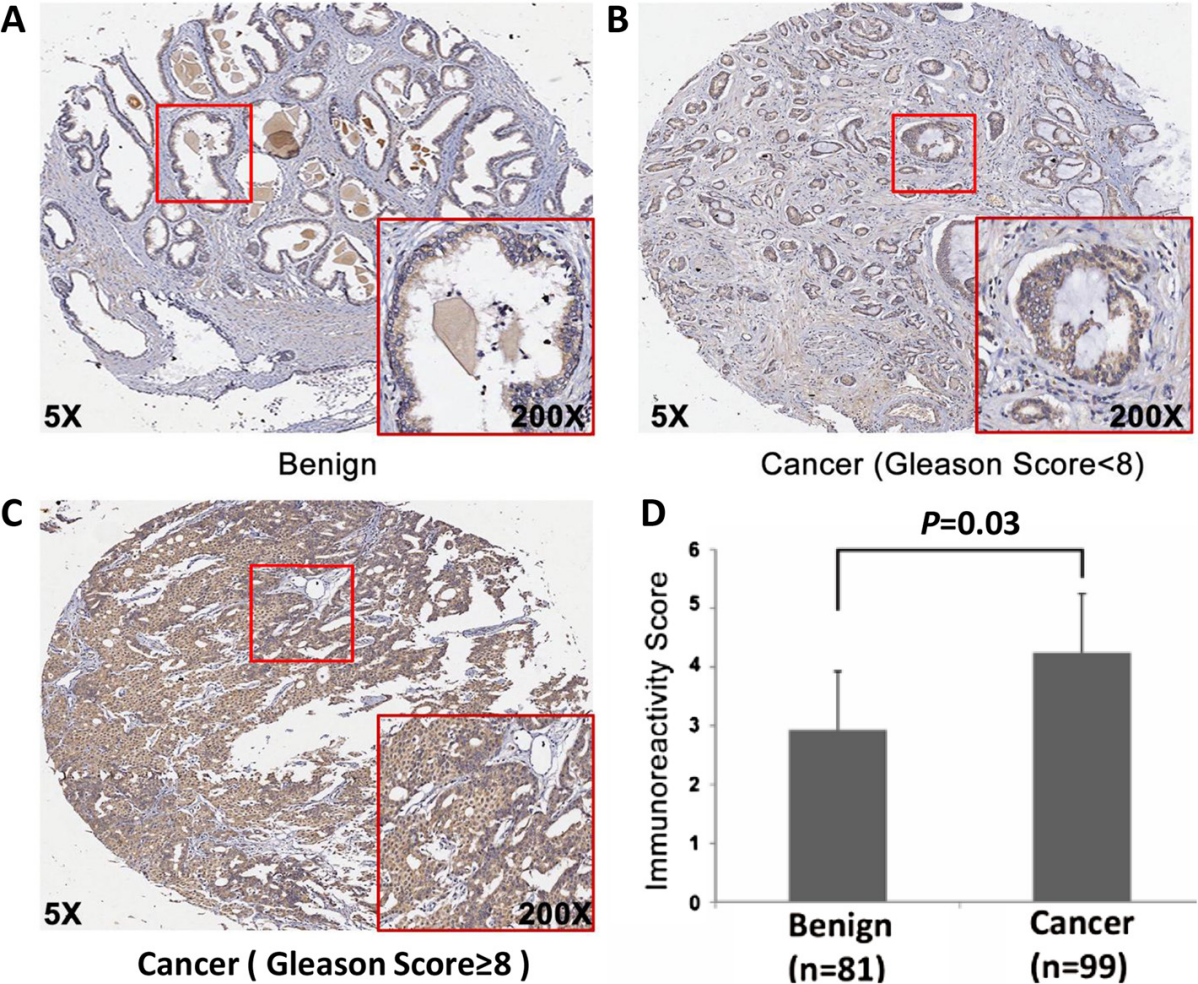
Immunohistochemical staining for NEK2 expression in PCa and adjacent non-cancerous tissues (original magnification × 50). **a** Benign prostate tissue. **b** PCa with a Gleason score <8. **c** PCa with a Gleason score ≥8. **d** statistical analyses showing higher immunoreactivity scores in cancerous tissues than in adjacent non-cancerous tissues. (IRS: Ca = 4.25 ± 1.36 vs Benign = 2.93 ± 1.51, *P* =0.03). **a**, **b**, and **c** were from TMA sample NO. A5, J8, and H8, respectively

We then analyzed the expression pattern and localization of NEK2 in a PCa TMA comprised of 99 PCa and 81 adjacent non-cancerous prostate tissues by immunohistochemistry staining. Representative pictures of immunohistochemistry staining of NEK2 are shown in Fig. 3a-c. NEK2 immunostaining was weak or undetectable in adjacent noncancerous prostate tissues (Fig. 3a). In contrast, strong staining of NEK2 was readily seen in the cytoplasm and nucleus of PCa tissues (Fig. 3b-c). A summary of NEK2 immunostaining in PCa tissues was shown in Table 2. Apparently, the PCa with a Gleason Score ≥8 had higher NEK2 expression than those with a Gleason Score <8. In addition, the PCa at high pathological stages showed increased NEK2 expression compared with PCa at low pathological stages. Moreover, statistical analyses of the Taylor dataset also showed that PCa with a Gleason Score ≥ 8 had higher expression of NEK2 than those with a Gleason Score <8 (*P* = 0.011) at the mRNA level. In addition, overexpression of NEK2 was also associated with metastasis, PSA failure, and overall survival time of patient, although no correlation between NEK2 expression at the mRNA level and pathological stages was seen in the Taylor dataset (Table 2).

**Table 2:** Association of NEK2 expression with the clinicopathological characteristics of prostate cancer

Clinical feature	IRS of NEK2 in our cohort			NEK2 expression in Taylor dataset		
	Case	$\overline{X}\pm s$	*p*	Case	$\overline{X}\pm s$	*p*
Age(years)						
<71 years	85	3.67 ± 1.43	0.904	146	7.54 ± 0.29	0.717
≥71 years	95	3.64 ± 1.70		4	7.48 ± 0.26	
Serum PSA						
<4 (ng/ml)	-	-	-	24	7.58 ± 0.32	0.440
≥4 (ng/ml)	-	-		123	7.53 ± 0.29	
Gleason score						
<8	70	3.81 ± 1.08	<0.001	117	7.48 ± 0.25	0.011
≥8	28	5.36 ± 1.39		22	7.71 ± 0.37	
Pathological Stage						
T2	70	3.81 ± 1.08	<0.001	86	7.48 ± 0.23	0.063
T3	29	5.31 ± 1.39		55	7.58 ± 0.35	
Metastasis						
No	99	4.25 ± 1.35	-	122	7.47 ± 0.25	<0.001
Yes	0	-		28	7.81 ± 0.33	
Overall survival						
Alive	-	-	-	131	7.51 ± 0.27	0.086
Die	-	-		19	7.68 ± 0.38	
PSA failure						
Negative	-	-	-	104	7.46 ± 0.25	<0.001
Positive	-	-		36	7.66 ± 0.31	

**Fig. 4:**
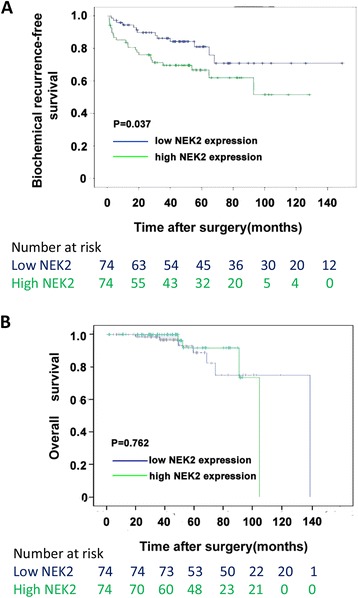
High NEK2 expression is linked to poor prognosis in PCa patients. **a** Kaplan–Meier analyses of the biochemical recurrence-free time of PCa patients with high NEK2 expression levels was shorter than those with low NEK2 expression levels (*P* = 0.037). **b** The overall survival time of PCa patients was not correlated to NEK2 expression levels (*P* = 0.762)

We then use the Kaplan–Meier method to analyze the association of NEK2 expression levels with the biochemical recurrence-free time and the overall survival time of PCa patients. The median of NEK2 expression in all PCa tissues of the Taylor dataset was used as the cutoff to divide all PCa tissues into high (*n* = 80) and low (*n* = 80) NEK2 expression groups. As shown in Fig. 4, the biochemical recurrence-free time of PCa patients with high NEK2 expression levels was shorter than those with low NEK2 expression levels. However, no correlation of the overall survival time of PCa patients with NEK2 expression levels was seen.

## Discussion

PCa affects one in nine men over the age of 65 and is the most frequently diagnosed cancer in American males [22, 23]. Early diagnosis through detection of serum prostate specific antigen (PSA) and improved procedures for surgical intervention and radiation therapy have significantly reduced the number of fatalities [24–27]. However, there is still no effective cure for men with advanced PCa that is often castration-resistant. Many studies have been dedicated to identifying prognostic markers that can be used to distinguish indolent versus aggressive forms of PCa. NEK2 is a centrosomal locating serine/threonine kinase required for centrosome duplication in mitosis. Expression of NEK2 is frequently upregulated in human cancers [9]. Recent evidence suggests that nuclear localization of NEK2 represents a strong predictor for drug resistance and poor prognosis in cancer [28]. Yet, the function of NEK2 in PCa progression is still obscure. Here, we reported the expression of NEK2 was elevated in PCa both at the mRNA and protein levels. As we just use one representative cell line for the study, the results could not completely reflect the relationship of NEK2 with androgen, which need further investigation to elucidate. The expression level of NEK2 was associated with the clinicopathological characteristics of PCa and tumor recurrence time of PCa patients. However, analyses of Taylor dataset revealed no statistical difference of NEK2 expression at the mRNA level in different pathological PCa group. Therefore, post translational modification of NEK is likely account for the differential expression of NEK proteins in normal prostate and PCa cells and tissues.

Aberrant NEK2 activity has been documented in multiple malignancies, including pediatric Ewing’s sarcoma, follicular lymphoma (FL), diffuse large B cell lymphoma (DLBCL), mammary gland tumor, leukemia, cervical, ovarian, breast cancers [10, 15, 16, 29–33].tumori Previous studies have shown that NEK2 contributes to assembly and maintenance of centrosomes and to bipolar spindle formation [12, 34, 35]. Therefore, inappropriately high expression of NEK2 might interfere with either centrosome integrity or chromosome segregation. However, the underlying cause for the increase in NEK2 expression is still unclear. This has led to a hypothesis that deregulation of centrosome function could be a major contributory factor to the genetic instability and loss of tissue differentiation that drive most cancer progression. Similar findings from other study indicate that aberrant expression and activity of NEK2 drive the progression and transformation of lymphomas that ultimately causes a more aggressive disease [31]. Specifically, overexpression of NEK2 in the ARP-1 multiple myeloma cells causes reduced treatment efficacy of bortezomib, doxorubicin, and etoposide [28]. Downregulation of NEK2 by shRNA inhibited myeloma cell growth and decreased drug resistance *in vitro* and in NOD-Rag/null gamma mice [19]. Paclitaxel and doxorubicin in combination with Nek2 siRNA or ASO treatment promote breast cancer cell apoptosis [36].

In summary, we reported that NEK2 was highly expressed in PCa cells and tissues. Depletion of NEK2 inhibited proliferation and tumorigenicity of the cells. In addition, expression of NEK2 was associated with poor outcome of PCa patients. Thus, the expression level of NEK2 has the potential for predicting PCa progression. A large study with more patient pool is warranted to determine whether expression of NEK2 can serve as a biomarker for PCa diagnosis and prognosis.

## Conclusions

The findings demonstrate that the overexpression of NEK2 is associated with progression in PCa and suggest that NEK2 has the potential to serve as a biomarker for PCa prognosis. Further validation with large sample pool is warrant.

## Abbreviations

NEK2: NIMA-related kinase 2
PCa: Prostate cancer
PSA: Prostate specific antigen
TMA: Tissue microarray
